# Oncoplastic Breast Surgery: Bridging Oncological Safety and Aesthetic Excellence in the Era of Precision Surgery

**DOI:** 10.7759/cureus.106404

**Published:** 2026-04-03

**Authors:** Shrikant Yadav, Harsh V Baranwal, Ram N Meena, Himanshu Panwar, Priya Kumari, Rahul Ranjan, Advitiya Jaswal

**Affiliations:** 1 General Surgery, Institute of Medical Sciences, Banaras Hindu University (BHU), Varanasi, IND; 2 General Medicine, Institute of Medical Sciences, Banaras Hindu University (BHU), Varanasi, IND; 3 Medical School, Institute of Medical Sciences, Banaras Hindu University (BHU), Varanasi, IND

**Keywords:** breast cancer, breast-conserving surgery, early breast cancer, oncoplastic breast surgery, therapeutic mammoplasty

## Abstract

Breast cancer is a significant health problem worldwide, being the most frequent cancer in females and one of the most lethal. The current approach to breast cancer has moved away from the morbid Halsted radical mastectomy to breast-conserving surgery (BCS). Although BCS, supplemented by radiotherapy, has the same survival rates as the Halsted procedure, it is associated with significantly good post-operative outcomes.

Oncoplastic breast surgery (OBS) combines the principles of breast cancer removal and reconstruction, allowing for the removal of larger tumors while maintaining the breast appearance by reshaping the breast tissue. In this article, the current evidence for the clinical, cosmetic, and oncological outcomes of OBS is reviewed. The evidence demonstrates the safety and potential benefits of OBS in comparison to BCS in the removal of breast cancer.

The article provides a background on the various classifications of OBS, including the evolution from volume-based to ptosis-based classifications by the American Society of Breast Surgeons. A comparison is made between volume displacement techniques, such as therapeutic mammoplasties, and volume replacement techniques, including regional perforator flaps and fat grafts. The article also discusses the challenges facing the adoption of OBS across the world, including the disparity in training between surgeons in developed and developing countries. Additionally, the article presents the challenges facing the adoption of OBS, including the risk of fat necrosis and fibrosis.

The article also presents the future direction in the development of OBS, including the use of extreme oncoplastic techniques in the removal of breast cancer using neoadjuvant chemotherapy. Additionally, the article presents the use of artificial intelligence in the pre-operative modeling of breast tissue using robotic-assisted tissue transfer. OBS is the current gold standard in the removal of breast cancer.

## Introduction and background

Breast cancer represents a significant public health challenge as it is the most prevalent cancer among women globally. According to GLOBOCAN 2022 projections, 2.3 million new cases and 666,103 deaths were anticipated in 2022. The lifetime risk of developing breast cancer is approximately 12.5% in Western countries and 13.5% in India and other developing nations [1,2]. Over the past century, the surgical approach has evolved from simple cancer excision to procedures that prioritise the preservation of form and identity. Halsted's radical mastectomy, while effective in reducing local recurrence, resulted in substantial disfigurement, lymphedema, mobility limitations, and psychological distress without improving survival outcomes [3]. In the mid-20th century, Bernard Fisher proposed that breast cancer is systemic from its onset, supporting the use of chemotherapy, hormone therapy, and breast-conserving surgery (BCS) [4]. Landmark studies such as Milan I and NSABP B-06 demonstrated that BCS with irradiation is equivalent to mastectomy for early-stage breast cancer [5].

BCS presents challenges in achieving clear margins while maintaining breast contour, often resulting in cosmetic deformities such as loss of glandular tissue, asymmetry, skin retraction, and nipple-areola complex (NAC) displacement. These issues are particularly prevalent in cases involving large tumours or tumours located in the central and lower poles. Additionally, irradiation may further compromise cosmetic outcomes. To address the psychosocial and physical morbidity associated with breast cancer treatment, a new surgical discipline has emerged that integrates oncologic safety with plastic and reconstructive techniques. Oncoplastic breast surgery (OBS) exemplifies this approach by utilising adjacent tissue mobilisation or volume replacement during tumour resection, thereby redefining success in breast cancer surgery [5,6].

## Review

Historical evolution of OBS

The concept of OBS originated in the late 1980s and early 1990s, when European surgeons recognised the limitations of lumpectomy [7]. The term “oncoplasty,” combining “onco” (tumour) and “plasty” (moulding), was first introduced by Werner Audretsch in 1998. Audretsch and collaborators in France, Italy, and the United Kingdom developed a multidisciplinary approach to OBS, integrating plastic surgery techniques with tumour resection. This innovation enabled large segmental resections that previously would have necessitated total mastectomy. OBS gained recognition as a distinct clinical entity following the Milan Consensus Conference on Breast Conservation in 2006, which emphasised that “the first requirement of OBS is that it must be absolutely oncologically safe, and aesthetic optimisation is of secondary importance” [8]. Over the past two decades, OBS has achieved widespread global acceptance, with utilisation rates in progressive European centres increasing from approximately 40% in 1991 to over 60% from 2002 onward, reflecting a shift toward holistic patient care and long-term survival [9]. OBS is now regarded as the definitive “third way” between conventional BCS and mastectomy [10].

Classification systems for oncoplastic breast surgery

With the proliferation of oncoplastic surgeries, it became essential to develop a universal classification (Table 1). Clough et al. proposed the first widely accepted classification, which classified oncoplastic breast surgeries into two levels depending on the amount of breast tissue removal and the complexity of reshaping [11]. Level I is removal of <20% breast volume, minimal skin removal, glandular mobilisation, and significant undermining in both the medial and lateral directions to reapproximate the tissues without any deformity. Level II is removal of 20-50% breast volume, requiring advanced therapeutic mammoplasty techniques, significant skin removal, NAC transposition on a vascular pedicle, and symmetrisation.

**Table 1:** Comparison of the foundational classification systems governing oncoplastic breast surgery. Source: This table is original and created by the authors based on references [11,12]. ASBS: American Society of Breast Surgeons

Classification System	Basis of Classification	Key Features	Clinical Utility
Clough et al. Classification [11]	Percentage of excised breast volume	Level I: <20% volume loss; simple glandular closure. Level II: 20–50% volume loss; requires therapeutic mammoplasty	Provides a simple, volume-based framework for surgical planning
ASBS Consensus Classification [12]	Anatomical ptosis and tissue availability	Volume Displacement: Rearrangement of native breast tissue (mastopexy, reduction). Volume Replacement: Use of autologous tissue (perforator flaps)	Emphasizes individualized, anatomy-based reconstruction and facilitates procedural standardization

Subsequently, the focus in OBS classification shifted from volume-based criteria to patient-specific anatomical factors, particularly native breast ptosis. In 2019, the American Society of Breast Surgeons (ASBS) established a consensus definition for OBS that de-emphasised volume considerations [12]. The ASBS defined OBS as "breast-conserving surgery including oncoplastic partial mastectomy with ipsilateral defect repair using volume displacement or volume replacement techniques, including symmetrisation as necessary." This definition facilitates practical repair processes following tumour excision, supports a team-based approach utilising algorithms, and aids in Current Procedural Terminology (CPT) coding for OBS in breast cancer management [13].

Preoperative evaluation and counselling of the patient

For OBS to be successful, a comprehensive preoperative evaluation is necessary. This is done in a multidisciplinary manner, including digital 3D tomosynthesis, high-frequency ultrasonography, and contrast-enhanced MRI to assess tumour margins, multifocality, multicentricity, and tumour relationship to regional vascular perforators.

The Six S's of preoperative evaluation of anatomy by surgeons include 1) Size: total volume, bra cup size; 2) Shape: degree of ptosis, desired post-operative shape; 3) Site: tumour location, particularly those in the medial, superior, and lower pole regions, where tumour resection is more challenging; 4) Skin: quality, redundancy, and proximity to tumour; 5) Symmetry: planning for contralateral surgery; and 6) Substrate: density of breast tissue, gland-to-fat ratios [14].

Indications for OBS include a tumour-to-breast volume ratio exceeding 20%, pre-existing macromastia or severe ptosis, and tumour locations prone to tethering or distortion of the NAC, such as central retroareolar, lower pole, or central lesions. OBS has been shown to improve quality of life by alleviating pain [15]. Absolute contraindications for OBS include a lack of guarantee of oncologic safety, including inflammatory breast cancer, where a radical modified mastectomy is required, and diffuse microcalcifications, where total gland removal is necessary. Relative contraindications for OBS include severe systemic conditions, including diabetes, autoimmune disorders, chronic tobacco use, and insufficient breast tissue for tumour removal, where total mastectomy is necessary [16].

Surgical techniques in oncoplastic breast surgery

OBS techniques (Table 2) can be classified into volume displacement and replacement, based on complex calculations [17]. 

**Table 2:** Comprehensive comparison of predominant volume displacement and volume replacement modalities utilized in oncoplastic breast surgery. Source: This table is original and created by the authors based on references [17-22]. LICAP: lateral intercostal artery perforator; AICAP: anterior intercostal artery perforator

Technique Type	Procedure	Indications	Advantages	Limitations
Displacement	Wise Pattern Mammoplasty	Large tumors (lower/central pole), macromastia	Allows large resections; improves breast shape	Long scars; risk of NAC necrosis
Displacement	Vertical Scar Mammoplasty (Lejour)	Moderate tumors, moderate ptosis	No inframammary scar; good contour	Limited for large resections
Displacement	Round Block (Benelli)	Small/periareolar tumors	Minimal scar; good cosmetic outcome	Risk of areolar widening
Replacement	Latissimus Dorsi (LD) Flap	Large defects, small breast volume	Reliable vascularity; large volume replacement	Donor site morbidity; scarring
Replacement	LICAP / AICAP Flaps	Lateral/lower pole defects	Muscle preservation; faster recovery	Technically demanding
Adjunct	Autologous Fat Grafting	Small defects, post-radiation deformity	Minimally invasive; improves tissue quality	Requires multiple sessions; variable resorption

Volume Displacement

In this technique, mobilization, rotation, and readvancement of original breast tissue are used to close the defect, similar to traditional techniques of reduction mammoplasty/mastopexy, but with lower donor-site morbidity. Wise pattern mammoplasty is a reliable technique for macromastia/ptosis, especially for large lower or central tumor defects [18]. A de-epithelialized dermal pedicle is used for NAC blood supply and repositioning, creating a new mound and a new scar pattern in the shape of inverted T/anchor. This technique is reliable for extensive tumor resection and functional, acceptable reduction. Vertical scar mammoplasty, including Le Jour, is preferred for moderate ptosis and mid-sized defects [19]. This technique allows a circumareolar incision that extends down to the inframammary crease without a horizontal Wise pattern. For small upper/periareolar tumour defects, the round block technique, also known as Benelli, is used. A window is created in the periareolar region by drawing concentric circles around the NAC. The dermis is excised between each circle, tumor resection is performed, and advancement is closed by a purse-string suture for camouflaging scars at the areola margin.

Volume Replacement Techniques

The extensive local excision causes a considerable defect that exceeds the replacement capacity of breast tissue. It is critical to replace the breast volume using regional or remote tissue to restore a natural breast shape rather than a flattened one. The traditional and conventional procedure for partial breast volume replacement is the latissimus dorsi (LD) myocutaneous miniflap procedure [20]. It is a flap procedure that includes a pedunculated flap from the LD muscle and a skin paddle. It is tunneled from the back through the axilla to replace the breast defect. Although this procedure is well-vascularized and effective for breast defect replacement, it has some disadvantages and morbidities related to the donor site, such as back seromas, extensive scarring on the back, and shoulder motion limitations. The modern microsurgical technique is based on chest wall perforator flaps that exclude muscle and minimize muscle-related morbidities. The lateral intercostal artery perforator (LICAP) and anterior intercostal artery perforator (AICAP) flaps are microsurgical procedures that include a fascia and skin flap supplied by a perforator vessel and a muscle group consisting of serratus anterior, external oblique, and rectus abdominis muscles [21]. It rotates a purely adipocutaneous flap to replace the breast defect without muscle resection.

Fat grafting is still a versatile option in reconstruction. By using low-pressure, atraumatic liposuction (usually abdominal) and micro-tunnel fat injection, irregularities are corrected and tissue quality is improved, especially after radiotherapy. Acellular dermal matrices (ADMs) are the most advanced options in partial volume replacement. They provide rapid tissue integration and high patient satisfaction rates (approximately 94%) without the need for further tissue harvesting [22].

Timing of reconstruction

The timing of reconstruction is crucial in the context of oncologic safety as well as aesthetics. Reconstructive timing is classified as immediate, delayed, and delayed-immediate (hybrid).

Immediate reconstruction is conducted simultaneously with tumor excision, offering substantial psychological and aesthetic benefits. However, it carries risks such as necrosis or wound breakdown, which may delay chemotherapy, and radiotherapy-induced fibrosis or contracture. Delayed reconstruction is performed months to years after tumor excision, thereby avoiding radiotherapy-related healing complications but increasing the risk of persistent body image concerns and challenges in achieving symmetry due to multiple surgeries and radiotherapy. Delayed-immediate reconstruction utilizes a temporary tissue expander during tumor excision, followed by adjuvant therapy and subsequent definitive reconstruction with implants or flaps. This approach minimizes cosmetic damage to the skin while ensuring uninterrupted oncologic treatment. Optimal timing should be determined using a personalised algorithm that incorporates patient and tumor characteristics [23].

Comparative oncologic efficacy and safety

One of the biggest concerns regarding OBS is that rearranging breast tissue could compromise oncologic safety. However, numerous studies involving tens of thousands of patients have consistently confirmed OBS oncologic efficacy [24]. Efficacy is defined as negative histologic margins, i.e., no invasive carcinoma or ductal carcinoma in situ (DCIS) at the inked margin. OBS offers wider negative margins without compromise of cosmetic results. This results in fewer positive margins and re-excision procedures than conventional BCS. OBS had a 2.5% positive margin rate versus 9.3% for conventional BCS [25]. A 2024 meta-analysis of 52 studies involving 46,835 patients found OBS superior to conventional BCS in terms of positive margins (RR 0.76), re-excision procedures (RR 0.68), completion mastectomies (RR 0.66), and local recurrence (RR 0.62) [26]. Moreover, long-term survival results confirm the safety of OBS, as local recurrence rates, disease-free survival, and overall survival rates are similar to conventional BCS and even mastectomies [27]. In addition, OBS is particularly successful in tumours larger than 4 cm or in patients with unfavourable tumour-to-breast size ratios, as demonstrated in reviews involving over 18,000 patients [28]. Five-year local recurrence rates in OBS patients range from 2.7 to 5.7% [29]. Clear margins in one procedure prevent delays in chemotherapy and radiotherapy. OBS ensures the quality of survivorship by protecting body image, sexuality, and overall well-being. Patient outcome is measured using standardized Patient Reported Outcome Measures (PROMs), where the most widely used PROM for satisfaction and quality of life in breast cancer patients is the BREAST-Q. Using systematic BREAST-Q data, it has been demonstrated that patients who undergo OBS have higher, long-lasting breast satisfaction compared to conventional BCS [30]. In addition, in studies using covariate analysis, unilateral mastectomies reduce the BREAST-Q by 21.3 points compared to conventional BCS, whereas the addition of oncoplastic techniques to conventional BCS improves the BREAST-Q by 9.2 points, thus proving the superiority of OBS in providing the best patient satisfaction after the procedure [30]. OBS provides patients with the best psychological and physical benefits by maintaining the original skin envelope, the inframammary fold, and the original breast symmetry, thereby reducing trauma and promoting psychosocial functioning, self-esteem, resumption of sexuality, exercise, and social activities. Aesthetic results in OBS patients are consistently reported to achieve 85%-95% in the evaluation of breast satisfaction [31].

Surgical complications and adjuvant radiotherapy in OBS

The procedures in OBS provide unparalleled oncological, aesthetic, and clinical benefits. However, the procedures in OBS are more anatomically and physiologically demanding than those in lumpectomy. OBS has a higher learning curve, higher requirements for interdisciplinary anatomy, and longer surgical times. It also has unique postoperative complications, which require sophisticated techniques and careful postoperative surveillance. The extensive mobilization of breast tissues in OBS, undermining in two planes, and long dermal pedicles increase the risk of local ischemia and venous congestion, resulting in minor complications with wound healing, seromas, and hematomas compared with BCS. Fat necrosis represents a significant challenge in OBS [32]. Mobilization or rotation of tissues can result in compromised microvascular supply, saponification, severe inflammation, and encapsulation in fibrosis. Fat necrosis manifests as hard, palpable, painful masses, creating concern for local recurrence among patients. It can also present radiologically with characteristics similar to malignancy or local recurrence, with microcalcifications, prompting the need to perform core needle biopsies to exclude recurrence or metastasis [33]. Adjuvant radiation therapy to the whole breast after any form of breast-conserving procedures adds physiological complexity to the OBS recovery process. High-dose radiation induces permanent microvascular changes and chronic inflammation, resulting in fibrosis, reduced elasticity, and volume loss [34]. In OBS procedures, tissue contraction may impair reconstruction symmetry, and in volume replacement with implants, radiation can cause capsular contracture. For optimal aesthetic outcomes, the surgeon must compensate for the volume loss due to radiation by overcorrecting [35]. Neoadjuvant chemotherapy (NACT) in locally advanced breast cancer (LABC) has revolutionized the treatment of breast cancer [36]. Previously, large tumor volumes in locally advanced breast cancer were treated with a morbid mastectomy to control the tumor volume [37]. However, the advent of powerful neoadjuvant chemotherapies has dramatically downstaged the tumor volume in LABC, resulting in a pathological response in some cases, even achieving a complete pathological response, thereby allowing breast-conserving therapy in the form of OBS [38]. The dramatic response of breast cancer to powerful neoadjuvant chemotherapies in conjunction with the development of advanced volume displacement/replacement procedures has resulted in the extreme form of breast-conserving procedures, namely, extreme oncoplastic breast-conserving surgery (eOPBCS), thereby widening the scope of BCS by allowing the removal of large tumor volumes, even in excess of 50% of the original breast volume, by means of therapeutic mammoplasty or perforator flap procedures [39]. According to recent meta-analysis studies, the overall survival and disease-free survival in patients undergoing the extreme form of oncoplastic breast-conserving procedures in locally advanced breast cancer with negative margins and effective systemic control are equivalent to total mastectomy procedures. However, the efficacy of the extreme form of oncoplastic breast-conserving procedures needs further evaluation in collaboration with medical oncologists, radiation oncologists, and oncoplastic surgeons [40].

Technological frontiers: AI and robotic-assisted microsurgery

OBS is undergoing significant technological advancements driven by AI, computer vision, and robotic-assisted surgical (RAS) technologies. These innovations aim to reduce errors, enhance intraoperative precision, and individualize surgical planning based on patient anatomy [41]. In preoperative planning, AI with deep learning is utilized to analyze 3D tomosynthesis and contrast-enhanced MRI scans for automated tumor segmentation, generating precise, rotatable 3D tumor maps. By integrating anthropometric data, breast volume, and ptosis, AI algorithms can simulate complex volume displacement and replacement procedures in a risk-free environment [42]. Intraoperatively, AI supports decision-making and tissue viability assessment. For microvascular reconstruction procedures such as deep inferior epigastric perforator (DIEP) and LICAP flaps, indocyanine green (ICG) fluorescence angiography is employed. AI-enhanced computer vision can quantify blood flow in real time and identify areas at risk for ischemia, thereby reducing fat necrosis and the need for reoperation [43]. RAS is transforming autologous tissue transfer by enabling minimally invasive incisions for DIEP and LD flaps, thereby reducing donor-site morbidity and recovery time. Additionally, robotic-assisted microsurgery can integrate machine learning data to provide real-time intraoperative guidance for microvascular anastomosis, potentially standardizing microsurgical outcomes globally [44]. Postoperatively, advanced AI frameworks can analyze extensive data on tumor biology, margins, genomics, and reconstructive factors to identify patients at highest risk for complications and facilitate proactive interventions [45].

## Conclusions

OBS has had a transformative and highly significant impact on the management of breast cancer in the 21st century. OBS integrates oncologic eradication with the preservation of female body image and psychological well-being. By combining the oncologic standard of wide excision with the precision of plastic surgical techniques, OBS has redefined breast cancer management and enhanced patient survivorship. A comprehensive evaluation of global clinical evidence indicates that OBS meets and often exceeds oncologic safety standards established by conventional BCS. OBS consistently achieves wider and clearer microscopic margins, reduces local recurrence rates, and lowers the need for re-operation. Additionally, OBS mitigates the psychological effects of surgical disfigurement by maintaining breast shape, natural contours, and inframammary crease. Restoration of symmetry between breasts results in aesthetically superior, natural outcomes associated with improved health-related quality of life, as measured by validated patient-reported outcomes such as the BREAST-Q. Despite these benefits, widespread adoption of OBS faces systemic barriers, including a steep learning curve that necessitates standardised interdisciplinary fellowship programs to ensure dual competence in oncologic resection and microvascular tissue transposition. High-volume clinical centres must also address the complication profiles of OBS, such as advanced fat necrosis and the complex interactions among autogenous tissue flaps, alloplastic devices, and high-dose adjuvant radiotherapy. Looking ahead, the therapeutic scope of OBS is expected to expand with standardised neoadjuvant downstaging in locally advanced breast cancer, enabling more extensive oncoplastic procedures. Furthermore, the rapid advancement and integration of artificial intelligence in microsurgery are poised to enhance preoperative analysis, microvascular precision, and individualised prediction of healing outcomes. As surgical techniques and associated technologies continue to evolve, OBS is positioned to become central to patient-centred breast cancer management and rehabilitation worldwide.
